# Prospective Multicenter Trial of Cervical Arthroplasty with the ROTAIO® Cervical Disc Prosthesis

**DOI:** 10.1177/21925682221109563

**Published:** 2022-08-05

**Authors:** Steffen Fleck, Anna Lang, Jens Lehmberg, Julia Fee Landscheidt, Ruediger Gerlach, Julian Rathert, Christian Ulrich, Ralph T. Schär, Sebastian Hartmann, Jan-Uwe Mueller, Claudius Thome

**Affiliations:** 1 Department of Neurosurgery, 60634 University Medicine Greifswald, Greifswald, Germany; 2 Department of Neurosurgery, Medical University Innsbruck, Innsbruck, Austria; 3 Department of Neurosurgery, Klinikum Bogenhausen, Munich, Germany; 4 Department of Neurosurgery, University Medicine Erfurt, Erfurt, Germany; 5 Department of Neurosurgery, Lindenhofspital Bern, Switzerland; 6 Department of Neurosurgery, Inselspital, Bern University Hospital, University of Bern, Switzerland

**Keywords:** cervical disc prosthesis, cervical arthroplasty, outcome, complications, adjacent segment disease

## Abstract

**Study design:**

Clinical observational study.

**Objective:**

The ROTAIO® cervical disc prosthesis is a novel unconstrained implant with a variable center of rotation aiming at physiological motion. The objective of this multicenter prospective trial was to evaluate clinical outcome and complications within 2 years.

**Material and Methods:**

120 patients (72 females and 48 males with median age of 43.0 years [23-60 yrs] underwent ACDA (ROTAIO®, SIGNUS Medical, Alzenau, Germany) and were prospectively followed for 24 months. Preoperative complaints were mainly associated with radiculopathy (n = 104) or myelopathy (n=16). There were 108 monosegmental and 12 bisegmental procedures including 6 hybrid constructs. Clinical outcome was evaluated at 3, 12 and 24 months in 100%, 96% and 77% of the cohort by VAS, NDI, WL-26, Patient`s Satisfaction Index (PSI), SF-36, Nurick Score, mJOA, Composite Success Rate, complications, patient`s overall satisfaction and analgesics use.

**Results:**

Highly significant clinical improvements were observed according to NDI and VAS (P < .0001 (arm); P < .001 (neck); P = .002 (head)) at all time points. Analgetic use could be reduced in 87.1 to 95.2%. Doctor`s visits have been reduced in 93.8% after 24 months. Patient`s overall satisfaction was high with 78.4 to 83.5% of patients. The composite success rate was 77.5% after 12 months and 76.9% after 24 months. There were no major complications in this series. Slight subsidence of the prosthesis was observed in 2 patients and 3 patients demonstrated fusion after 24 months. 2 patients developed symptomatic foraminal stenosis, so that implant removal and fusion was performed resulting in a revision rate of 1.7% in 2 years.

**Conclusion:**

The ROTAIO® cervical disc prosthesis is a safe and efficient treatment option for symptomatic degenerative disc disease demonstrating highly significant clinical improvement and high patient`s overall satisfaction with very low revision rates at 2 years.

## Purpose

Since its introduction in the 1950s^1^ Anterior Cervical Discectomy and Fusion (ACDF) has become a standard surgical procedure for the treatment of cervical disc disease in patients with radiculopathy or myelopathy. ACDF is performed to achieve neural decompression, segmental stabilization and to maintain cervical lordosis. ACDF yields good clinical outcome and high fusion rates^2,3^. However, fusion sacrifices the mobility of the operated segment leading to increased biomechanical forces at the level of the non-fused adjacent segments und this may accelerate Adjacent Segment Disease (ASD).^4,5^ Anterior Cervical Discectomy and Arthroplasty (ACDA) has been introduced as an alternative that preserves segmental mobility of the operated segment aiming at decreasing the risk of ASD. Although recent randomized clinical trials^6-10^ have compared both techniques, this issue is still controversially debated^4,11-14^ and mid-to long-term results are sparse. Currently, there are many disc prostheses with different biomechanical properties commercially available, which claim to imitate physiologic motion. Standard ball and socket designs and (semi-) constrained devices, however, don´t allow uncoupled translation and are thus thought to force the facet joints into non-physiologic movements. As this may interfere with successful outcome over time, our group focused on an unconstrained disc prosthesis with uncoupled translation.^15,16^

The aim of this multicenter, multinational prospective observational study was therefore to evaluate clinical outcome and complications with the newly developed artificial cervical disc prosthesis ROTAIO® (SIGNUS Medizintechnik GmbH, Alzenau, Germany) within a follow-up period of 24 months. Thus, the aim and design of our study was not a two-armed comparison to other disc arthroplasty devices. Nevertheless, we wanted to relate our results and the features of the used prosthesis to other devices in the market.

## Material and Methods

The study complied with the Declaration of Helsinki and was approved by all local ethics committees of the involved centers (initial approvement: Ethics Commission, Medical University Innsbruck, Austria). All patients gave written informed consent.

### Patient population

Consecutive patients meeting the inclusion criteria were prospectively included from July 2014 to January 2019.

### Inclusion criteria

(1) age >18 and <60 years,

(2) Degenerative Disc Disease (DDD) at one or two levels between C3/4 to C6/7 and a disc height of at least 50% in comparison to other segments,

(3) no improvement of clinical symptoms after at least 6 weeks of conservative treatment or significant or progressive neurological deficits at the time of presentation,

(4) a minimum NDI of 15 points (30%)

#### Exclusion criteria

(1) cervical instability demonstrated on flexion/extension radiographs
(2) kyphotic index segment
(3) non-mobile index level and/or increased osteochondrosis in index disc/facet joints
(4) previous surgery of the cervical spine

### Device Design

**Figure 1.**
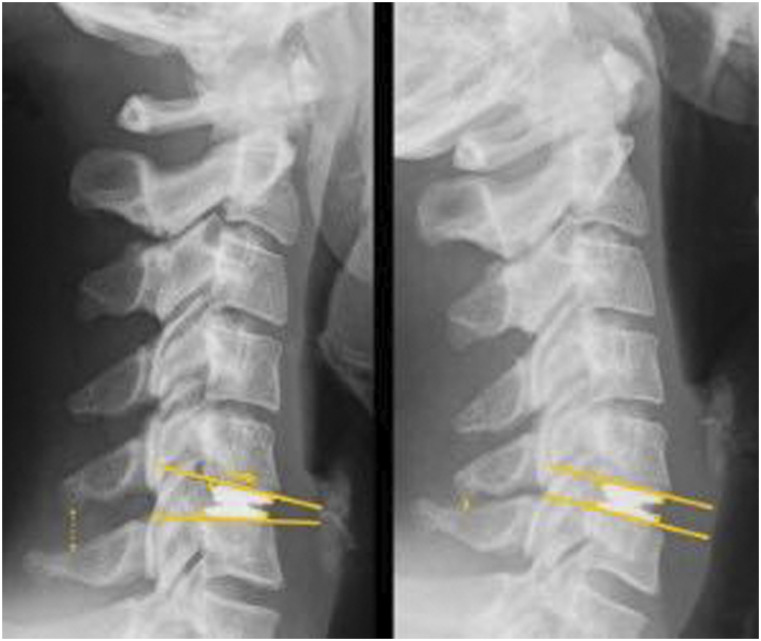
The ROTAIO® cervical disc prosthesis allows uncoupled anterior translation upon flexion and posterior translation upon extension mimicking natural disc motion.

The ROTAIO® cervical disc prosthesis (Figure 1) is used to replace the disc after anterior cervical discectomy. The aim of disc replacement is to restore disc height and to maintain physiological motion of the index segment. The ROTAIO® prosthesis is a new unconstrained implant with a variable center of rotation (COR) and uncoupled translation to enable physiological facet joint-guided motion. The prosthesis consists of a superior and an inferior endplate (Titanium alloy to ISO 5832-3), on which the sliding elements (Cobalt-Chrome alloy to ISO 5832-12) are anchored and secured by means of a fixation pin. Primary stabilization is achieved via toothed surfaces of the endplates, which are additionally blasted to increase surface area and allow rapid bony integration.

### Surgical Procedure

Surgery was performed by board-certified neurosurgeons who had attended manufacturer`s instruction lectures. A standard anterior cervical approach with discectomy was used including microsurgical resection of the posterior longitudinal ligament, parts of the uncinate process and visualization of the nerve roots as indicated. After application of the sizing trials, the prosthesis was implanted under fluoroscopic control in a cage-like fashion strictly according to the company`s instructions during slight distraction of the intervertebral space. The single piece arthroplasty device is implanted in a cage-like fashion without specific milling of the endplates.

### Clinical evaluation

All patients were scheduled for clinical examination on the day before surgery, postoperatively prior to discharge, and 3, 12 and 24 months after surgery. In addition to a clinical examination including neurological status validated self-assessment outcome measures were used: Visual Analogue Scale (VAS) (range 0-10) for head, neck and arm pain separately, Patient`s Satisfaction Index (PSI), Neck Disability Index (NDI) (range 0-50), Work Limitation Questionnaire (WL-26), and Quality of Life Questionnaire (SF-36).^17,18^ The Nurick-Score for classification of gait disturbance and the Modified Japanese Orthopaedic Association Score (mJOA) were recorded. Complications related to the implant, fusion of the index level and surgical procedures at the index level (revision surgery) or at an adjacent level (for ASD) were also documented. A Composite Success Rate was defined as the combination of (1) improvement in NDI (≥15%) compared to preoperatively, (2) stable or improved neurological status compared to preoperatively, (3) no secondary operation, (4) no implant-associated complication.

### Imaging protocol

Plain anteroposterior and lateral radiographs in neutral position and lateral radiographs in flexion and extension were obtained in each patient. Furthermore, all patients underwent magnetic resonance imaging before surgery. Computed tomography was additionally applied at the discretion of the surgeon.

### Statistical Analysis

Data are presented as the mean value ± standard deviation (SD) of the mean.

The trial was designed to detect an absolute difference between pre- and postoperative data. Comparisons were performed with the use of an unpaired t-test, or in case of nonparametric values with the Wilcoxon-Mann-Whitney-U test. Furthermore, x^2^ was used for differentiation of categories.

Differences were considered statistically significant for two-tailed *P*-values <.05 (power of 80%, maximum dropout rate of 20%). Data analysis was performed with IBM SPSS Statistics Vers. 20.0.

## Results

### Demographic Data

120 patients (60% female, 40 % male) with a median age of 43.0 years (range: 23 to 60 years) were included. The majority of patients underwent ACDA at the C5/6 (58.8%) and the C6/7 (49.6%) levels. Preoperative complaints were mainly associated with radiculopathy (n=104; 86.7%) or myelopathy (n=16; 13.3%). There were 108 monosegmental and 12 bisegmental procedures including 6 hybrid constructs comprised of ACDF with cage fusion and ACDA with the ROTAIO® disc prosthesis. Minimum follow-up of 12 months was available in 115 patients and 92 patients had completed 2-year follow-up.

### Clinical evaluation

The median duration of symptoms amounted to 5 months. Demographics and preoperative symptoms are listed in Table 1. There were some differences between radiculopathic (RP) and myelopathic (MP) patients.

**Table 1.** demographics and preoperative symptoms.

	%	x^2^ (p)	Radiculopathy (RP)	Myelopathy (MP)	overall
Age (median) (yrs)		.1273	42.7 ± 8.4	39.2 ± 7.5	43 (23-60)
Female	60.0				
Male	40.0				
Leading symptoms (RP vs. MP)			87%	13%	
ASA (I-II)	80.7				
ASA (III)	19.3				
VAS overall		.1120	5.9 ± 2.4	4.7 ± 2.0	
Daily analgetics	84	.0479	87%	67%	
doctor`s visits (≥ monthly)	51				
Physiotherapy		.0577	68%	30%	
Alcohol		.0225	51%	11%	
Smoking	42				

Highly significant clinical improvements were observed for **VAS** arm (P<.0001), neck (P<.001) and head (P = .0022) at all time points. Table 2 **Significant functional** improvements were observed with a ≥15% decrease in **NDI** in ≥90% at 12 and 24 months after surgery. Differences of RP and MP are noticed in Table 3.

**Table 2.** clinical results (focusing on visual analogue scale (VAS) and patient`s satisfaction index (PSI).

	preoperative	3 months	12 months	24 months
VAS overall (0-10)	6.5 ± 2.2	− 4.5 ± 2.8	− 4.2 ± 3.1	− 4.4± 2.7
VAS head (0-10)	3.0 ± 2.8	− 1.6 ± 2.6	− 1.3 ± 2.9	− 1.0 ± 3.1
VAS neck (0-10)	5.7 ± 2.4	− 3.8 ± 2.5	− 3.7 ± 2.9	− 3.4 ± 2.7
VAS arm (0-10)	6.1 ± 2.6	− 4.4 ± 3.1	− 4.1 ± 3.0	− 4.3 ± 3.0
PSI (satisfied vs. nonsatisfied)	-	83.5% vs. 4.1%	78% vs. 7%	79% vs. 7%

**Table 3.** functional outcome.

	Radiculopathy (RP) (%)	Myelopathy (MP) (%)	RP and MP (%)	Significance (p) (χ^2^)
12 months				
NDI (decrease ≥ 15%)	92.6	70	90.0	.02
Strength and mJOA (stable or improved)	91.4	100	92.5	.34
complication	6.3	9.1	6.6	.75
Composite success rate	80	60	77.5	.16
24 months				
NDI (decrease ≥ 15%)	93.0	75.0	90.8	.10
strength And mJOA (stable or improved)	96.5	100.0	96.9	.59
complication	8.9	7.7	8.7	.99
composite Success rate	78.9	62.5	76.9	.30

The neurological status according to muscle **strength** and **mJOA** remained stable or improved in 92.5% and 96.9% respectively. The **composite success rate** was 77.5% after 12 months und 76.9% after 24 months Table 4.

**Table 4.** complications (summarized after 24 months).

Complication (summarized after 24 mths)	n (of 92)	%
Major complication	0	0
Transient approach related	2	2.2
Intermittent/transient cracking noises	2	2.2
Slight subsidence of prosthesis	2	2.2
Fusion after 24 months	3	3.3
Revision at index level/foraminal stenosis	2	2.2
Operation at adjacent level (24 mths)	0	0

Analgesic medication could be reduced after 3 months in 91.3%, after 12 months in 87.1% and after 24 months in 95.2% of patients, but increased in 8.8%, 12.9% and 4.8%, respectively. Preoperatively, the analgesics consumption was significantly higher in the RP group than in the MP group (see Table 1). As expected, improvement and success rate tended to be more pronounced in the RP group.

The **WL-26** clearly demonstrated a reduction of work limitations (P < .0001 at 3, 12 and 24 months). Health-related Quality of Life (**SF-36**) revealed a highly significant improvement (P < .0001) for the following items within 3, 12 and 24 months: body function, social function, psychologic well-being, physical pain, vitality, and overall health perception.

**Patient’s overall satisfaction** was high after 3, 12 and 24 months with 83.5%, 78.4% and 79.1% of patients, while 4.1%, 6.8% and 7.0% respectively were not satisfied. **Doctor’s visits** for cervical spine problems have been reduced in 93.8% after 24 months and increased in 6.2%.

### Complications

There were no major complications in this series. Temporary morbidity related to the anterior cervical approach but not the implant per se, like recurrent nerve palsy and significant dysphagia, occurred in 2 patients. Initially, 2 patients experienced intermittent and transient cracking noises. Slight subsidence of the prosthesis was observed in 2 patients and 3 patients demonstrated fusion after 24 months. 2 patients developed clinical problems associated with foraminal stenosis after 3 and 9 months, respectively, so that implant removal and fusion was performed. Overall, total complication rate amounted to 9.2% and revision rate to 1.7% at the index level and no procedures at the adjacent levels within 2 years. No revision was needed due to the prosthesis itself.

## Discussion

Anterior Cervical Discectomy and Fusion (ACDF) is a standard procedure for the treatment of degenerative cervical disc disease. Based on the notion that preserving motion reduces the risk of Adjacent Segment Disease (ASD),^19,20^ Anterior Cervical Discectomy with Arthroplasty (ACDA) has been introduced as an alternative to fusion in the 1990^′^s. Although clinical outcome is well documented for both techniques,^4,11^ some patients will experience persistent or increasing symptoms over time.

### Adjacent Segment Disease

Despite its well documented benefits, ACDF may cause ASD in mid- and long-term follow-up.^13,21-25^ Biomechanical studies have shown increased intradiscal stress and motion compensation in the levels adjacent to the fusion site^26,27^ with a change of the center of rotation in adjacent levels postoperatively.^28,26,29^ Although this is considered by some authors to be the underlying cause for ASD, it is still controversial if this is attributable to the biomechanical effects of fusion or to the natural history of cervical degeneration.^4,14,30^

Reoperation due to ASD has been documented at a rate of 2.9% annually after ACDF.^31^ ACDA is considered as an alternative to ACDF preserving normal cervical kinetics and biomechanics.^32^ Thus, the rate of additional surgeries may be reduced with less stress on adjacent levels using ACDA.^33,34^ The pooled surgery rate for ASD after disc prosthesis (ACDA) was 3.8% (.9 -7.6%) within a follow-up of up to 84 months summarizing 13 randomized controlled trials (RCT).^35^ Although clinical short-term results are satisfactory,^7,9,21^ there are only a few studies reporting mid-to long-term results.^36-39^ Garrido et al^37^ reported improved functional outcome for ACDF and ACDA on 24 and 48 months follow-up with no degradation of the outcome measures between 2 and 4 years after surgery. This is in concordance with the results of Goffin et al,^38^ who reported consistent if not improved clinical results at 4- and 6-years follow-up compared to the 1- and 2-years postoperative results. Our study supports these data that patients improved significantly after surgery and the clinical results remained stable on mid-term follow-up.

The protective effect of ACDA on the adjacent discs remains controversial. In the single level arm of their prospective cohort study, Kim et al.^25^ observed ASD in 13% of all patients treated with ACDA compared to 23% in the ACDF group at a median follow-up of 19 month. Walraevens et al. reported ASD in the adjacent upper and lower segment to the operated site for up to 8 years after ACDA.^39^ Similar observations were made by Ding et al.^36^ They observed mild ASD in the adjacent levels in approx. 23% of all patients. The degeneration mainly manifested as new formation or enlargement of an anterior osteophyte. However, no degeneration in clinical outcome occurred due to the lack of a direct relation between radiographic and clinical ASD.^36^ In our cohort with the new ROTAIO® prosthesis, no patient required adjacent level surgery within 2 years.

### Quality of motion

After ACDA, emphasis is often placed on presence and magnitude of motion as assessed by ROM, while quality of motion by parameters like instantaneous COR, COR, and instantaneous axis of rotation has just recently been identified as important for evaluating changes in the cervical motion pattern.^40-43^ Anderst et al. demonstrated that the instantaneous COR was generally fixed in the longitudinal direction, but it translated in the anterior-posterior direction during flexion-extension.^42^ If translation is not adequately possible, non-physiologic stress on the facet joints at the index level ensues, which may cause facet joint syndrome, as it has commonly been seen in lumbar disc arthroplasty. Liu et al. evaluated the instantaneous COR located at the superior half of the lower vertebral body height and the posterior half of its width, and changing with age.^44^ It has been postulated that these further findings should be considered in clinical practice and when designing disc prostheses.^40^

Although the overall effectiveness of ACDA has already been demonstrated, the kinematic properties of the various designs differ substantially.^15,45^ The Bryan disc prosthesis with its almost unconstrained design retained kinematic motion adequately,^46-48,49,50^ yielding a near-physiological rotation at the index level.^51^ Ball-and socket designs like the Prestige LP (semiconstrained design) and the Prodisc-C (semiconstrained with fixed axis of rotation), however, did not fully restore normal mobility in view of ROM and COR, which may cause secondary problems over time.^16^ Particularly neck pain can be an ongoing problem after ACDA as a result of abnormal forces and load on the facet joints.

The ROTAIO® cervical disc prosthesis has been designed to allow physiologic facet-guided motion by enabling uncoupled translational movement. While some other modern disc prostheses may also harbor mechanisms to achieve uncoupled motion, e.g. by deformation of elastic material, this is completely uncoupled and unhindered in the device used in our study.

The current knowledge on motion of cervical disc prosthesis has been nicely summarized by Shin et al.^52^ While ball-in-trough devices are capable to allow some translational motion, ball-in-socket devices are not. The spectrum of devices includes constrained, semiconstrained and unconstrained prostheses depending on their range of motion.^53^ Constrained or semiconstrained devices tend to shift the COR anteriorly and/or superiorly, while an unconstrained prosthesis tends to maintain the preoperative location of the COR.^54^ The ROTAIO® cervical disc prosthesis stands out in its properties, as both anterior-posterior as well as mediolateral translation is truly uncoupled from flexion-extension and/or lateral bending, thus allowing the motion to be guided by the facet joints in an unrestricted fashion. While motion in non-translational and/or (semi-)constrained devices may interfere with successful outcome over time particularly in the long-term due to facet joint problems, this should not be the case with the prosthesis used in this study.

The low revision rate and the stable clinical results over time in this series seem to support these considerations.

### Clinical Outcome

In 2016, preliminary clinical and radiographic results with the ROTAIO® cervical prosthesis demonstrated excellent results.^55^ Our present results with more than 100 patients in a multicenter prospective trial confirm these findings with highly significant clinical improvement and high patient`s overall satisfaction. Pain relief, reduction of analgesics consumption, functional improvement, reduction of disability, patient satisfaction and quality of life were found to be very high and at least comparable to previous IDE trials. The increase in analgesics use in a small percentage of patients after surgery may partly be explained by myelopathy as primary complaint preoperatively and routine analgesic regimens postoperatively. Revision rate was very low and no implant failure was observed. No surgical procedure due to ASD was performed within 2 years.

Nevertheless, longer follow-up is necessary to prove durability and functionality of the prosthesis. In view of our current data, however, the ROTAIO® prosthesis is a suitable alternative to ACDF and other available prostheses. The particular biomechanical characteristics with uncoupled translation and a variable center of rotation may allow physiological cervical spine motion with low fusion and low ASD rates.

Although numbers are low and no statistical analysis was performed, all patients with hybrid constructs performed well within 24 months. This subgroup should be addressed in specific studies in the future, as hybrid solutions may offer an alternative to multilevel fusion procedures.

### Hybrid Constructs

Although our patient number with hybrid constructs is low and no valid conclusions can be drawn from these results, bisegmental hybrid solutions are an important aspect of cervical arthroplasty. Increased motion above an ACDF may impose biomechanical stress on an arthroplasty device, which may lead to hypermobility and/or late failure. Hur et al. have compared the unconstrained ROTAIO prosthesis with a semi-constrained device in bisegmental hybrid constructs in a total of 82 patients with 2-year follow-up.^56^ There was better neck pain improvement, C2–C7 ROM recovery and less impact at the superior adjacent level with the ROTAIO prosthesis. Mobility in the arthroplasty segment remained stable in the ROTAIO group, while it increased over time with the semi-constrained device. This seems to indicate that an unconstrained device, which does not force the facet joints into artificial motion, may also be beneficial for hybrid solutions. However, further long-term studies on hybrid constructs are mandatory.

### Limitations

This prospective observational multicenter study of consecutive patients has received research support by the manufacturer, although clinical data was assessed and analyzed by the investigators. The study was not intended to compare the ROTAIO® results to ACDF or other prostheses. Follow-up rates were 96% at 1 year and 77% at 2 years, which may limit the validity of the results. Nevertheless, loss to follow-up is comparable to previous trials and IDE studies. The follow-up is currently limited to 2 years with some loss of follow-up and must thus be considered intermediate, so that long-term sequelae cannot yet be adequately monitored.

## Conclusion

The ROTAIO® cervical disc prosthesis with its unconstrained design with uncoupled translation and a variable center of rotation is a safe and effective treatment option for symptomatic degenerative disc disease. Highly significant clinical improvement, high patient`s overall satisfaction, and very low revision rates after a follow-up of 24 months could be demonstrated in this prospective, observational study.

## Appendix

### Abbreviations

ACDA: anterior cervical discectomy and arthroplasty
ACDF: anterior cervical decompression and fusion
ASD: adjacent segment disease
COR: center of rotation
DDD: degenerative disc disease
FSU: functional spinal unit
MP: myelopathy
NDI: neck disability index
n.s: not significant
NSAID: non-steroidal anti-inflammatory drugs
PSI: patient’s satisfaction index
RCT: randomized clinical trial
ROI: region of interest
RP: radiculopathy
SF-36: short form -36
VAS: visual analogue scale
WL-26: work limitation
QoL: quality of life
